# The Pore-Forming Haemolysins of *Bacillus Cereus*: A Review

**DOI:** 10.3390/toxins5061119

**Published:** 2013-06-07

**Authors:** Nalini Ramarao, Vincent Sanchis

**Affiliations:** INRA, UMR 1319 MICALIS-AgroParisTech, La Minière, Guyancourt 78280, France; E-Mail: vincent.sanchis@jouy.inra.fr

**Keywords:** *B. cereus*, haemolysins, cereolysin O, haemolysin II, haemolysin III, cytotoxin K, PlcR

## Abstract

The *Bacillus cereus sensu lato* group contains diverse Gram-positive spore-forming bacteria that can cause gastrointestinal diseases and severe eye infections in humans. They have also been incriminated in a multitude of other severe, and frequently fatal, clinical infections, such as osteomyelitis, septicaemia, pneumonia, liver abscess and meningitis, particularly in immuno-compromised patients and preterm neonates. The pathogenic properties of this organism are mediated by the synergistic effects of a number of virulence products that promote intestinal cell destruction and/or resistance to the host immune system. This review focuses on the pore-forming haemolysins produced by *B. cereus*: haemolysin I (cereolysin O), haemolysin II, haemolysin III and haemolysin IV (CytK). Haemolysin I belongs to the cholesterol-dependent cytolysin (CDC) family whose best known members are listeriolysin O and perfringolysin O, produced by *L. monocytogenes* and *C. perfringens* respectively. HlyII and CytK are oligomeric ß-barrel pore-forming toxins related to the α-toxin of *S. aureus* or the ß-toxin of *C. perfringens.* The structure of haemolysin III, the least characterized haemolytic toxin from the *B. cereus*, group has not yet been determined.

## 1. Introduction

The *Bacillus cereus sensu lato* group contains diverse Gram-positive spore-forming bacteria that are widespread in the environment. This group comprises seven closely related species: *B. mycoides*, *B. pseudomycoides*, *B. weihenstephanensis*, *B. anthracis*, *B. thuringiensis*, B. cytotoxicus and *B. cereus sensu stricto* [1]. Comparisons of genome sequence similarity between species have highlighted the close relationships between these bacteria, making their identification to species level difficult. Contrary to *B. subtilis*, which is considered as a true soil saprophyte, the metabolic potential of *B. cereus* species is poorly suited for the degradation of plant material, or to metabolize a range of complex carbohydrate polymers. Indeed, the functions of the genes for complex carbohydrate metabolism seems to be limited to the degradation of glycogen, starch, chitin and chitosan, all of which are important components of insect tissues. Furthermore, *B. cereus* appears to contain many protease genes and many peptide and amino-acid transporter genes. Together, these observations indicate that *B. cereus* is better adapted to a protein diet and that its primary source of nutrients is probably animal tissues [2].

A new genetic structure of the entire *B. cereus* group has recently been proposed. This phylogenetic taxonomy separates the strains into seven groups (I to VII), principally on the basis of their ability to grow at various temperatures [1]. However, not all the bacterial species within a given genetic group have the same capacity to induce disease, and the distinction between pathogenic and innocuous strains is far from clear, for the entire *B. cereus* group [3]. Moreover, the genetic determinants of *B. thuringiensis* and *B. anthracis* pathogenicity are located on plasmids, which can be exchanged between the various group members, transforming *B. cereus* into *B. thuringiensis* or *B. anthracis* through simple plasmid acquisition. Other than these specific plasmid genes, the genomes of the three species, *B. anthracis*, *B. thuringiensis* and *B. cereus,* are very similar, and the genetic determinants required for non-species-specific aspects of infection may be common to all the bacteria of the *B. cereus* group [2,4].

*B. cereus sensu stricto*, or *B. cereus* as it is usually called*,* is an emerging human pathogen that causes gastroenteritis and is now considered the third most important cause of collective food poisoning incidents in Europe, after *Salmonella* and *Staphylococcus aureus* [5]. In 2008, 102 confirmed outbreaks of food borne disease caused by *B. cereus* were identified by the European community, corresponding to more than a thousand patients [5]. However, *B. cereus* food borne illness incidents are probably largely under-reported, as the reporting of *B. cereus* food borne poisoning is not mandatory. *B. cereus* causes two types of food borne illnesses. In both types, the symptoms usually last less than 24 h, but several fatal cases of bloody diarrhoea and *emetic* poisoning have been reported, mainly in older and debilitated persons [6,7,8,9,10,11]. The “short-incubation” or emetic form, with symptoms similar to those of *S. aureus* infections, is caused by the ingestion of cereulide, a peptide produced by the bacterium and already present in the ingested food [12]. This small molecule is believed to bind to 5-hydroxytryptamine 3 (5-HT3) receptors causing vomiting and acts like an ionophore leading to inhibition of mitochondrial activity. The “long-incubation” or diarrhoeal form, which resembles the food poisoning caused by *Clostridium perfringens*, is characterized primarily by abdominal cramps and diarrhoea, following an incubation period of 8 to 16 h [13]. This type of the disease is generally associated with the ingestion of bacteria producing toxins [14,15]. 

Although *B. cereus* is generally considered to be mainly associated with gastrointestinal disorders and severe eye infections, it is also an opportunistic human pathogen associated with a multitude of other local and systemic infections such as parodontitis, necrotising infections, endocarditis, nosocomial acquired bacteremia, osteomyelitis, sepsis, liver abscess, pneumonia and meningitis, particularly in postsurgical patients, immunosuppressed individuals, intravenous drug abusers and neonates [16,17,18,19]. However, despite the increasing frequency with which such non gastrointestinal diseases are being reported, there is still little recognition and appreciation of the role of *B. cereus* in these serious, and frequently fatal, clinical infections in humans.

In early stationary phase, *B. cereus* produces several compounds (degradation enzymes, cytotoxic factors and cell-surface proteins) that might contribute to virulence [20,21,22,23,24,25,26], and the illnesses associated with this organism are probably mediated by the synergistic effects of a number of virulence products. These products, known to accumulate only during stationary phase when high bacterial densities are reached, include two enterotoxic complexes (haemolysin BL (HBL) and non-haemolytic enterotoxin (NHE)), several phospholipases-C, a collagenase and various haemolysins/cytolysins (HlyI, HlyII, HlyIII and HlyIV) [13].

**Figure 1:**
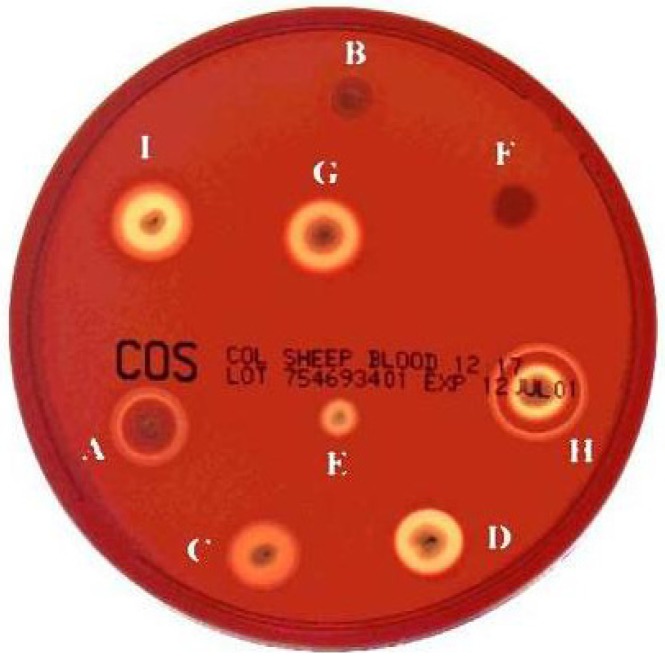
Haemolytic types (HT) for *B. cereus* and *B. thuringiensis* strains isolated from small soil samples. Haemolytic activity was determined at 30 °C on sheep blood agar plates. All 198 strains were plated on blood agar at the same time and compared after 15 h. The haemolytic activity of each strain was estimated twice, and each replicate was classified “blind” with respect to the previous one*.* Nine different haemolytic types (A to I) were identified among the 198 strains [31].

Most of these proteins are active against erythrocytes, and could therefore be defined as haemolysins and most *B. cereus* and *B.* *thuringiensis* strains form large, but distinct haemolytic halos when grown on human or sheep blood agar plates (Figure 1). HBL and NHE are homologous three-component pore-forming toxins inducing cell lysis in various eukaryotic cells [7,27]. The crystal structure of HblB and the modeled 3D structure of the other components show homology to the 3D structure of the pore-forming haemolysin cytolysin A (ClyA) from the Gram-negative enteric pathogen *Escherichia coli* [28]. HBL displays a unique ring-shaped haemolytic type (HT) in gel diffusion assays (haemolytic type H in Figure 1). NHE was originally described as having no haemolytic activity, accounting for its designation as “non-haemolytic” enterotoxin. However, NHE has also been shown, more recently, to be haemolytic towards erythrocytes from several mammalian species [28]. HBL and NHE have already been described and reviewed in detail elsewhere [13,29], and will not be addressed in this review. 

*B. cereus* may also cause haemolysis by the combined/synergistic action of phosphatidylcholine-specific phospholipase C (PC-PLC) and sphingomyelinase (SPH), which form a biological complex known as cereolysin AB (CerAB) [30]. This membrane-disrupting complex specifically hydrolyses sphingomyelin in the intact erythrocyte membranes, causing erythrocyte haemolysis. The molecular properties of cereolysin AB result in haemolysis through enzymatic degradation of the cell membrane, but not by pore formation, and are therefore, beyond the scope of this review.

This review will therefore focus on the four other pore-forming haemolysins produced by *B. cereus*: haemolysin I or cereolysin O, haemolysin II, haemolysin III and haemolysin IV or CytK, as it is more commonly known. 

## 2. Haemolysin I (Cereolysin O)

### 2.1. Genomic and Structural Features

**Figure 2:**
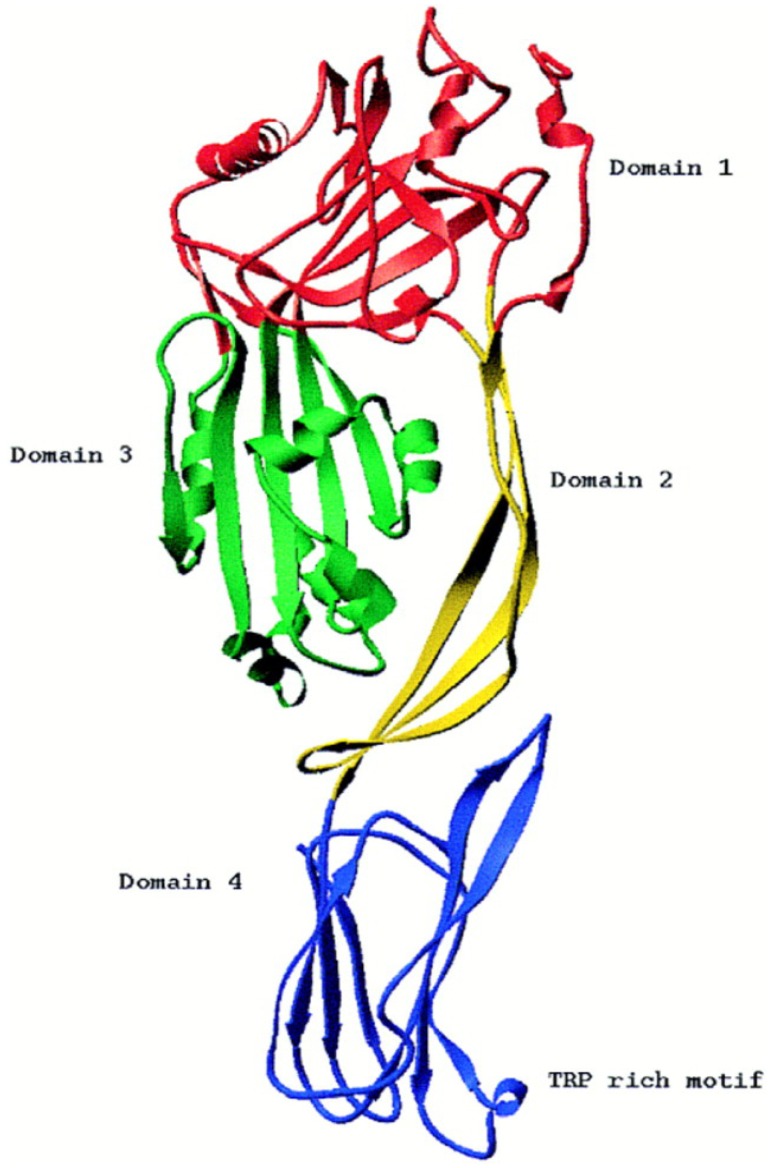
Structure of the Perfringolysin O molecule shown in ribbon representation. Reproduced with permission from [40].

Haemolysin I was first described in 1967 [32], and was referred to as cereolysin O (CLO) in *B. cereus*, thuringiolysin O (TLO) in *B. thuringiensis* and anthrolysin O (ALO) in *B. anthracis* [33]. These proteins are very similar, with 98% identical amino-acid sequences [34]. CLO is a heat-labile protein whose haemolytic activity is inhibited by cholesterol (<10 μg/mL) and neutralized by anti-streptolysin-O globulins from hyperimmune horse serum [32,35]. CLO also shows 57%–68% amino acid similarity to perfringolysin-O (PFO) and streptolysin-O from C*lostridium perfringens* and *Streptococcus pyogenes*, respectively [36]. Haemolysin I, produced by *B. cereus*, can therefore be considered to belong to the cholesterol-dependent cytolysin (CDC) family (formerly known as thiol-activated cytolysins) and much about the structure and function of this molecule can be inferred from information available for the other members of the family [36]. There are a number of detailed reviews on this toxin family that consists of over 25 members that are produced by many different species of Gram-positive bacterial pathogens [37,38]. These toxins consist of a single polypeptide chain with a molecular weight of 50 kDa to 80 kDa. The amino-acid sequences of the proteins of this family have an overall pairwise sequence identity of 40% to 70% that is distributed relatively evenly over the entire sequence of each molecule, suggesting they all have similar activities and 3D structures. These toxins also contain a highly conserved tryptophan-rich sequence (ECTGLAWEWWR) of 11 residues (an undecapeptide) in domain 4, close to the *C*-terminus, which participates in the binding of some CDCs to cholesterol-rich membranes [39]. The CDC toxins disrupt cell membranes, by forming large pores, up to 30 nm in diameter. In each case, the cytolytic activity of the CDC specifically requires the presence of cholesterol in membranes and does not seem to depend on any other specific cell-surface receptor. CDCs are, therefore, able to lyse the cytoplasmic membranes of almost all animal cells [37]. The entire crystal structure of a soluble monomer of PFO, the prototype of this toxin family, was determined in 1997 to a resolution of 2.7 Å (Figure 2) and shown to be a β-sheet-rich, four-domain protein [40]. The monomer is folded into four discontinuous domains, forming an elongated mushroom-shaped molecule. The crystal structure of a second member of the CDC family, the intermedilysin (ILY), secreted by *Streptococcus intermedius*, has also been determined to a resolution of 2.6 Å [41], and more recently, the crystal structure of the soluble state of ALO was solved, in a pre-pore conformation, to a resolution of 3.1 Å [42]. The structural comparison of PFO, ILY and ALO revealed that the three-dimensional structures are well conserved and that the three proteins adopt a similar characteristic four-domain architecture, in which domain 4 is involved in membrane recognition, domain 3 is involved in β-sheet insertion, and domain 2 is the hinge region that undergoes a large conformational change. The putative membrane-binding domain 4 comprises the last 110 *C*-terminal residues, including the conserved tryptophan-rich undecapeptide. In addition, three other short hydrophobic loops, L1, L2 and L3, flanking the undecapeptide at the tip of domain 4, have been shown to insert into the membrane surface and to anchor the CDC to the membrane [43]. The structure and molecular mechanism of several other CDCs are now also relatively well characterized. Pore forming by CDCs involves oligomerisation and the assembly of soluble monomers into a ring-shaped pre-pore, which undergoes a conformational change for insertion into the membrane, to form a large amphipathic transmembrane β-barrel structure [44]. 

### 2.2. CLO in Virulence

CDCs are cytolytic proteins produced by diverse Gram-positive bacteria. They can lyse or permeabilize host cells or intracellular organelles during infection. CDCs can also lyse various types of cells, including erythrocytes, *in vitro*. The best known members of this family are listeriolysin O, perfringolysin and pneumolysin O, produced by *Listeria monocytogenes*, *C. perfringens* and *S. pyogenes*, respectively. For *L. monocytogenes*, which releases its listerolysin O into host phagocytic cells, this protein has been shown to be important for virulence and essential for cellulosome escape and the intracellular multiplication of the bacterium [45]. In extracellular human pathogens, such as *C. perfringens*, which releases perfringolysin O into the extracellular environment, the toxin disrupts plasma membranes, causing cell death by necrosis. In *B. anthracis*, the recombinant ALO (rALO) purified from *E. coli* is extremely active against human erythrocytes [33]. In 2006, Mosser and Rest showed that ALO killed human monocytes, neutrophils, macrophages and lymphocytes, providing support for the notion that ALO is one of the virulence factors of *B. anthracis* [46]. *B. cereus* CLO is lethal when injected intravenously (1–2 μg) into mice and is haemolytic at concentrations as low as 1 ng/mL [32]. CLO also induces the release of lactate dehydrogenase from retinal tissue *in vitro*, suggesting that it may exacerbate the necrosis typical of *B. cereus* endophthalmitis [47]. More recently, Bourdeau *et al*. showed that the apical application of ALO decreased the barrier function of human polarized gut epithelial cells as well as increased intracellular calcium, which caused specific rearrangement of the tight junction protein occludin [42]. Given that *B. anthracis* spores can enter the host through the gastrointestinal (GI) route, and that the epithelial cell barrier is one of the major obstructions to infection in the GI tract, they proposed that ALO-induced increase of intracellular calcium to alter tight junction architecture could lead to movement of the vegetative anthrax bacteria, or other bacterial toxins, into the surrounding tissues. However, additional research is needed to evaluate the validity of this hypothesis. The haemolysin I of *B. cereus sensu lato* therefore seems to perform various functions during bacterial infection.

### 2.3. Gene Regulation

**Figure 3:**
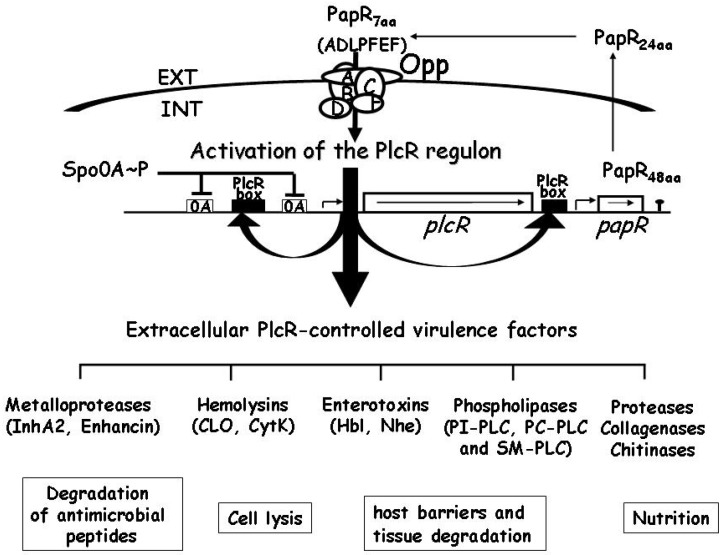
Schematic representation of the transcriptional regulator PlcR and its cognate cell-cell signalling peptide PapR. The activity of PlcR depends on PapR, a secreted signalling peptide re-imported into the bacterial cell through the Opp system [51]. After its export, PapR is cleaved to a *C*-terminal heptapeptide active fragment that accumulates in the medium [52]. When high bacterial densities are reached, PapR concentration increases inside the bacterial cells, promoting its interaction with PlcR. The PapR-PlcR complex then binds to its DNA recognition site, the palindromic PlcR box, triggering a positive feedback loop that upregulates the expression of a regulon of 45 genes encoding proteins that are essentially secreted or bound or attached to cell wall structures at the interface between the bacterial cell and its environment [55]. These proteins are likely to be involved in host tissue degradation or in protecting the bacterial cell from host immune defenses, and may act together to provide food supply. Ultimately, when the bacteria enter the sporulation process, *plcR* transcription, and consequently PlcR-regulated gene expression, is repressed by the sporulation key-regulator Spo0A.

In *B. cereus* and *B. thuringiensis*, the expression of CLO and TLO is controlled by the transcriptional activator PlcR [48,49]*.* PlcR was originally identified as a transcriptional activator of *plcA*, the gene encoding phosphatidylinositol-specific phospholipase C [50]. For activity, PlcR requires PapR, a peptide expressed as a propeptide under the control of PlcR, which is exported out of the cell, processed to generate an active heptapeptide, and re-imported into the bacterial cell through the OppABCDF oligopeptide permease system (Figure 3) [51,52]. The PlcR/PapR system is now known to be the central transcriptional regulator for virulence genes in *B. cereus* at the onset of the stationary phase [53,54]. It controls the expression of a large regulon comprising at least 45 genes, including degradation enzymes, cell-surface proteins and toxins, most of which may be considered potential virulence factors [55]. 

In *B. anthracis*, the PlcR regulon is silent due to a nonsense mutation in the *plcR* gene that inactivates the PlcR protein. *B. anthracis* is, therefore, generally considered to be non-haemolytic. However, Shannon *et al*. demonstrated that ALO is expressed under certain growth conditions, suggesting that haemolysin I regulation in *B. anthracis* is different from that in *B. cereus* and *B. thuringiensis* [33]. However, the nature of the regulatory mechanisms and conditions promoting ALO expression in *B. anthracis* remain unknown.

## 3. Haemolysin II

### 3.1. Genomic and Structural Features

**Figure 4:**
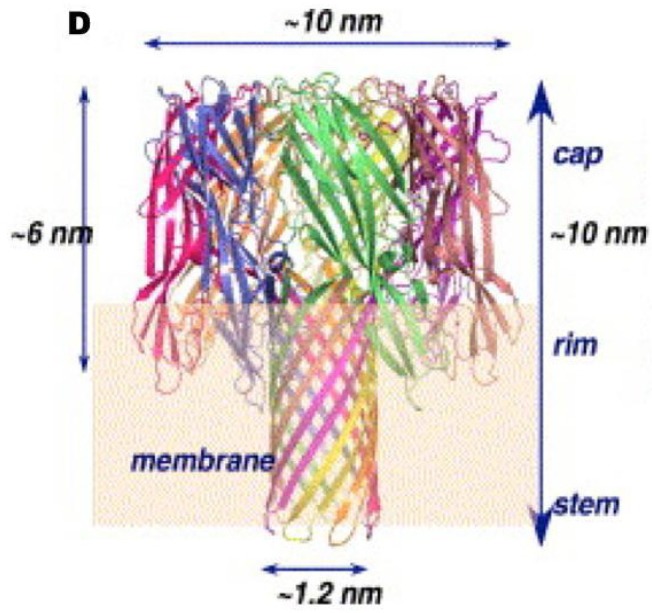
Molecular model of the *B. cereus* haemolysin II heptameric pore. Molecular modeling was performed using the 3D structure of α-hemolysin from *S. aureus* [60].

In addition to CLO, early studies detected at least one *B. cereus* haemolysin, which was called haemolysin II (HlyII) [56]. HlyII activity is generally unaffected by cholesterol or anti-streptolysin O antibodies [57]. It is heat-labile and susceptible to proteolytic enzymes. The predicted 412-amino acid sequence of the HlyII precursor is reduced to a mature size of 381 amino acids (42.6 kDa) and this protein has a pI of 8.56 [58]. HlyII is an oligomeric ß-barrel pore-forming toxin. This group of toxins includes the α-toxin of *Staphylococcus. aureus*, the ß-toxin of *C. perfringens* and the *B. cereus* cytotoxin K (CytK) [59,60]. HlyII displays 28%–31% amino-acid sequence identity to the staphylococcal α-toxin, but it also has a 94-amino acid *C*-terminal extension, absent from the other known members of the β-barrel pore-forming toxin family, which is not required for pore formation or haemolytic activity [61,62]. These β-barrel pore-forming toxins bind to membranes and form heptameric oligomers. Each monomer inserts a glycine-rich segment into the membrane to form the walls of a transmembrane pore. HlyII also contains a glycine-rich segment, suggesting a similar mechanism of membrane penetration, and has been shown to forms anion-selective channels with functional diameters of about 7 Å in planar lipid bilayers (Figure 4) [59,60,61,62]. HlyII induces erythrocyte lysis, although it has been reported that HlyII has no specific receptor in erythrocytes [59]. In addition, HlyII induces the lysis of phagocytic cells (insect haemocytes, mouse macrophages, human monocytes and dendritic cells) but not epithelial cells [63,64]. The related α-toxin of *S. aureus* appears to bind to the host cell receptor phosphocholine [65,66] allowing the protein to accumulate locally in microdomains enriched in cholesterol and sphingolipids (lipid rafts). This suggests that certain cell types have high-affinity toxin-binding sites favouring toxin oligomerisation and thus stable membrane-anchored binding to target host cells [65]. The ß-toxin from *C. perfringens* has also been shown to concentrate in lipid rafts [67]. 

### 3.2. Haemolysin II in Virulence

HlyII is haemolytic and cytotoxic to human cell lines [59], but has not been implicated in the diarrhoea caused by *B. cereus.* It has been suggested that its lack of contribution to diarrhoea results from a trypsin digestion site in the ß-loop constituting the transmembrane domain of the toxin, resulting in inactivation by trypsin in the small intestine [8,13], as observed for the ß-toxin of *C. perfringens* [68], but this remains to be tested experimentally. It has recently been shown that HlyII accumulates in high oxidation-reduction potential (ORP) conditions, but not in low ORP conditions, suggesting that the regulation of HlyII secretion is redox-dependent [69]. As anoxic and low ORP conditions mimic the intestinal environment, HlyII is unlikely to be a virulence factor involved in gastrointestinal disease. However, it has been shown that HlyII is involved specifically in immune cell death by apoptosis [62,63] and that it is produced in large amounts by clinical strains of human origin [15], strongly suggesting a role in opportunistic infections.

Non-gastrointestinal *B. cereus* infections are characterized by bacteremia, despite the accumulation of inflammatory cells at the site of infection [70], implying that the bacteria have developed a means to resist the activity of inflammatory cells and thus, of the host immune system. *B. cereus* can circumvent the host immune response, as *B. cereus* spores survive, germinate and multiply in contact with macrophages [71], eventually leading to the production of secreted toxins responsible for host cell death [63]. HlyII has been shown to be one of the toxins responsible for host cell death [63,64]. Indeed, HlyII has haemolytic properties against a broad range of erythrocytes [59]. Its haemolytic activity with rabbit blood cells is more than 15 times more potent than that of *Staphylococcus* α-toxin [59,62]. In addition, HlyII induces pore formation in the membranes of various eukaryotic cells [59,60,61], and the apoptosis of host monocytes and macrophages *in vivo*, in a death receptor-dependent pathway (activation of caspase 8) [63]. The ability of HlyII to kill macrophages may account for the persistence and dissemination of *B. cereus* in the host. The induction of apoptosis by *B. cereus* may cause tissue damage and compromise the antimicrobial immune response, thereby promoting bacterial spread, leading to the associated signs and symptoms of disease. Expression of the *Bacillus cereus hlyII* gene in *B. subtilis* induces haemolysis and virulence in a crustacean infection model [72]. HlyII has also been shown to be involved in *B. cereus* virulence in insects and mice [62]. The important role of HlyII has been highlighted by the presence of the *hlyII* gene in several clinical isolates of *B. cereus* [15], although its distribution remains limited [73]. 

### 3.3. Regulation of Expression of the *hlyII* Gene

HlyII is one of the few secreted virulence factors of *B. cereus* that is not regulated by PlcR, the central transcriptional regulator for virulence genes in *B. cereus*, which is required for the transcription of *hbl*, *nhe*, *clo* and *cytK*. Instead, it has been shown to be down-regulated by the specific transcriptional regulator HlyIIR [74,75] and by the global regulator Fur [76,77]. The *hlyII* and *hlyIIR* genes are present in the same chromosomal locus (Bc 3523 and Bc 3522, respectively, in the ATCC 14579 strain) but are not organized into an operon. *In vitro* analyses have shown that recombinant HlyIIR regulates *hlyII* expression by the specific binding of two dimers to a perfect 44 bp inverted DNA repeat (22 bp × 2) centred 48 bp upstream from the *hlyII* transcription initiation site [78]. The crystal structure of HlyIIR contains a large internal hydrophobic cavity that could accommodate a ligand with a molecular mass of up to 500 Da, suggesting that cofactor-dependent activation is involved in the inhibition of *hlyII* expression [79]. It has been suggested that the binding of the appropriate ligand to HlyIIR could change the orientation of the DNA-binding domains, modifying the affinity of the ligand-bound form for the specific DNA site [79]. Consistently, glucose-6-phosphate, a carbon source that is commonly used and consumed by bacteria during their growth [80], binds directly to HlyIIR, increasing its capacity to bind to its DNA box upstream from the *hlyII* gene, thereby inhibiting its expression [75]. 

HlyII expression is also regulated by the ferric uptake regulator (Fur). The *hlyII* promoter contains a Fur binding site overlapping the transcription start site [81] and the binding of Fur to this Fur box competes with RNA polymerase binding to the *hlyII* promoter, thus interfering with *hlyII* expression [77]. The deletion of *fur* in *B. cereus* results in lower virulence in an insect infection model, demonstrating a link between virulence and iron metabolism [81]. It has consistently been shown that the *hlyII* gene is down-regulated by iron during bacterial infection [77].

**Figure 5:**
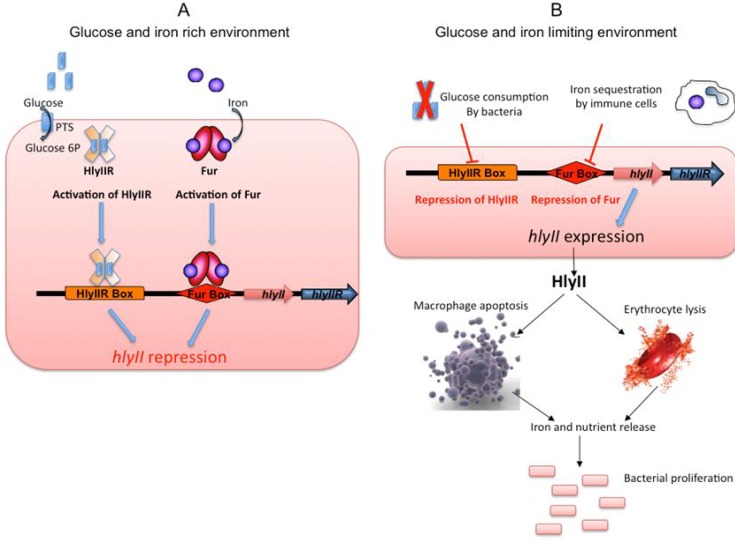
Model of the role and expression of *hlyII* during infection (**A**) As long as iron and glucose are abundant in the bacterial environment, glucose enters the bacteria as glucose 6P (blue rectangles) and binds HlyIIR (plain orange cross). Iron (purple circles) binds Fur (red ovals). These binding events promote the repressor activities of HlyIIR and Fur, leading to the HlyIIR- and Fur-based transcriptional repression of *hlyII* gene expression (**B**) By contrast, when glucose and iron become scarce, *hlyII* expression is activated. HlyII is then released into the environment and induces macrophage and erythrocyte lysis. The dead cells release their intracellular content, providing access to metabolites that are essential for bacterial growth [75].

Sugar and iron are crucial compounds for bacterial multiplication and, thus, for their capacity to colonise their hosts. As a model, we suggest that, when glucose is consumed by the bacteria and iron is sequestered by phagocytic cells as a natural host defence [82,83], the HlyIIR and Fur repressors become inactivated and *hlyII* expression is triggered. HlyII is then produced by the bacteria and secreted, triggering the death of haemocytes and macrophages [63]. The contents of the cells are then released into the environment, providing the bacteria with access to nutrients and allowing them to grow, and thus promoting a new cycle of *hlyII* gene inhibition/expression (Figure 5). 

## 4. Haemolysin III

### 4.1. Genomic and Structural Features

Haemolysin III (HlyIII) is the least characterized haemolytic toxin from the *B. cereus* group. Its 657-nucleotide gene has been cloned and characterized in *Escherichia coli*. It is a heat labile protein whose haemolytic activity is not inhibited by cholesterol. The presumed precursor has a molecular weight of 24.4 kDa and a pI of 9.42. The toxin has not been purified, and it is unknown whether it is secreted by members of the *B. cereus* group. Its amino-acid sequence contains no obvious signal peptide for secretion [84]. However, membrane topology prediction using the TMpred program predicts that it does contain six 19- to 22-residue transmembrane segments typical of an integral membrane protein [85]. Moreover, 55% of the protein consists of contiguous non-polar residues, suggesting that it would be unlikely to remain soluble in aqueous phase. Sequence analysis of the promoter region of *hlyIII* does not reveal the presence of the highly conserved palindromic region (TATGNAN_4_TNCATA) that is the specific recognition target for PlcR activation indicating that expression of *hlyIII* is PlcR independent.

### 4.2. HlyIII in Virulence

The role of HlyIII in virulence has not been investigated *in vivo* and remains a matter of speculation. However, HlyIII induces a haemolytic phenotype on human blood agar when expressed in the heterologous host *E. coli* [86]. Osmotic protection experiments with *E. coli* lysates as the source of haemolysin have suggested that HlyIII forms transmembrane pores with an estimated diameter of 3–3.5 nm [86]. The toxin forms oligomeric pores in three steps: protein binding to the erythrocyte surface, monomer assembly to form the transmembrane pore, which finally leads to erythrocyte lysis. Binding and pore formation seem to be temperature dependent, whereas the lysis of erythrocytes is not [86].

## 5. Haemolysin IV (Cytotoxin K)

### 5.1. Genomic and Structural Features

Haemolysin IV, or CytK, is a 34 kDa cytotoxic necrotic and haemolytic protein that was first partially characterized by Beecher *et al*. in 2000 [87]. The protein was then isolated, cloned and sequenced from the NVH 391/98 strain [8]. This strain was responsible for a severe food-borne outbreak of diarrhoeal disease in which several elderly people had bloody diarrhoea, three of whom died [8]. Four other similar strains have since been isolated, also mostly from food poisoning cases. These strains have been shown to form a robust and well separated cluster in the *B. cereus* group. Strain NVH 391/98 is now the type strain of a newly validated species of the *B. cereus* group, *Bacillus cytotoxicus* [88]. 

The deduced amino-acid sequence of the protein is 37% identical to that of *B. cereus* haemolysin II described above and 30% identical to that of the α-hemolysin from *S. aureus*, suggesting that, from a structural point of view, CytK (like HlyII) belongs to the family of oligomeric β-barrel pore-forming toxins [8]. The *S. aureus* α-haemolysin is one of the best characterized, pore-forming toxins. The structure of the pore created by a heptamer of this toxin has been determined to a resolution of 1.9 Å [89]. These toxins are secreted in a soluble form, which is eventually converted into a transmembrane pore by the assembly of an oligomeric β-barrel, with the hydrophobic residues facing the lipids and the hydrophilic residues facing the lumen of the channel [90]. In 2004, Fagerlund and coworkers isolated, from *B. cereus* strain NVH 1230/88, a variant of CytK (CytK-2) with an amino-acid sequence 89% similar to that of the first CytK isolated (CytK-1). The differences between the CytK-1 and CytK-2 proteins were clustered in certain regions of the protein [91]. The strains harboring CytK-1 have recently been defined as a new species, *B. cytotoxicus* (see above), different from the *B. cereus* strains harboring the CytK-2 variant [92]. Both CytK proteins possess a Sec-type signal peptide, suggesting that CytK secretion occurs via the Sec translocation pathway. Consistently, intracellular accumulation and low levels of secretion of the toxin have been reported in cultures supplemented with sodium azide, an inhibitor of the SecA pathway [93].

### 5.2. CytK in Virulence

Little is known about the underlying mode of action of CytK at the cellular level. *In vitro* studies have revealed that this toxin forms pores that are weakly anion selective in pure phospholipid planar bilayers. The predicted minimum pore diameter for cytK is about 7 angströms [94], but the stoichiometry of oligomers and the structure of the pores remain unknown. Moreover, CytK can spontaneously form oligomers that are resistant to SDS but not boiling, a feature of other β-barrel pore-forming toxins. CytK-1 has been shown to be highly toxic to Vero and Caco-2 cells [94]. This suggests that the mode of action of this toxin involves the formation of pores in epithelial cells, causing fluid release and the destruction of the epithelial cells, resulting in necrosis [94]. The more common and more recently described variant of CytK, CytK-2, is also haemolytic and toxic to human intestinal Caco-2 cells and Vero cells. However, the CytK-2 protein from *B. cereus* NVH 1230/88 is only about a fifth as toxic to human intestinal Caco2 cells and Vero cells as the CytK-1 variant originally isolated from strain NVH 391-98 [91]. Both the CytK-1 and CytK-2 proteins form pores in planar lipid bilayers, but most of the channels observed with CytK-2 are of lower conductance than those created by CytK-1. Both variants of the CytK toxin are likely to contribute to the enterotoxicity of *B. cereus* strains. As *B. cereus* is not a homogeneous species, the food poisoning potential of a particular *B. cereus* strains is probably determined by a combination of adaptation traits, virulence factors and expression levels of toxin genes in a given strain [95,96].

### 5.3. Distribution of cytK Genes in *B. cereus*

The occurrence of *cytK* in strains of *B. cereus* has been investigated. In 2002, Guinebretière *et al.* analyzed 37 food-poisoning and 25 food-borne *B. cereus* strains for the presence of *cytK* and the gene was found in less than 50% of the strains; *cytK* was detected in 73% of *B. cereus* isolates involved in diarrhoeal disease, but in only 37% of isolates from food not implicated in disease [97]. Similarly, in 2006, Wijnands *et al*. reported that the *cytK* gene was present in almost 50% of the 796 isolates tested, which originated from 182 different food samples [98]. In another recent study on 411 *B. cereus* and 205 *B. thuringiensis* strains, from food and soil, *the cytK* gene was detected in 365 (89%) of the *B. cereus* and 172 (84%) of the *B. thuringiensis* strains [99]. In 2006, a PCR-based detection system was developed and tested to provide insight into the distribution of *cytK*-1 and *cytK*-2 in the *B. cereus* group [92]. In a total of 391 *B. cereus*, from various phylogenetic groups (I to VII), most of the strains positive for *cytK* harbored the *cytK-2* variant, the *cytK-1* form being specific for the most distant group VII “*B. cytotoxicus*”. The *cytK-2* form was particularly frequent in mesophilic groups III and IV, whereas it was rare or absent from the psychrotolerant or moderately psychrotolerant groups (VI, II, and V). The *cytK* gene was absent from group I strains [100]. 

### 5.4. Regulation of cytK Expression

*cytK* gene expression is regulated by the quorum sensing PlcR/PapR system [55,95]. Sequence analysis of the promoter region of plcR-regulated genes has revealed the existence of a highly conserved palindromic region that acts as the specific recognition target for PlcR activation [55]. The *cytK-1* promoter region of *B. cereus* strain NVH 391-98 contains a putative PlcR box, TATGCAATTTCGCATA, diverging from the defined consensus recognition site, TATGNAN_4_TNCATA (the underlined nucleotides are the most highly conserved in all the PlcR boxes), as initially defined for PlcR-controlled genes. In *B. cereus* strain ATCC 14579, the transcription of *cytK-1* starts at the onset of the stationary phase and peaks two hours later, as is the case for PlcR-controlled genes. Moreover, the expression of *cytK-1* is shut off in a PlcR-deficient mutant, indicating that its transcription is PlcR-dependent and that the PlcR box in the *cytK-1* promoter region is functional, despite a mismatch in the PlcR recognition site. As the *B. cereus* NVH 391-98 strain has been shown to synthesize larger amounts of *cytK1* mRNA than other *B. cereus* strains [101], it is even possible that this mismatch in the plcR box accounts for the higher level of *cytK-1* expression in *B. cytotoxicus*. By contrast, another *B. cereus* strain, NVH 883/00, carrying the same *cytK-1* gene displays very low levels of *cytK-1* expression and is not cytotoxic [102]. This suggests that variations in cytotoxicity between strains may be determined by differences in the level of expression of the toxin genes rather than by the presence of one or other of the two forms of the toxin or sequence polymorphism between the toxins. The *cytK*-1 promoter regions in these strains were identical and the expression of PlcR was similar [101] indicating that differences in genetic backgrounds, between strains harbouring otherwise identical promoter and *cytK* gene sequences, may result in differences in *cytK* gene expression, leading to differences in CytK production. However, the exceptionally high level of *cytK-1* expression of *B. cytotoxicus* NVH391-98 remains unexplained and may be due to as yet unidentified regulatory mechanisms [102]. The expression of *cytK* in *B. cereus* was recently investigated by developing a fluorescent *B. cereus* ATCC 14579 reporter strain containing the cyan fluorescent protein (CFPopt) gene under control of the *cytK* promoter. The authors found that toxin production was similar in the reporter and wild-type strains. However, only a small proportion of the reporter cells (1%–2%) displayed fluorescence, and thus *cytK* expression, suggesting that toxin production originated from a small subpopulation of *B. cereus* ATCC 14579 in a homogeneous monoculture [103]. It remains unclear whether these results are specific to the ATCC14579 strain or can be generalized to a larger number of strains of different origins. Further studies are therefore required to unravel the complex regulatory pathway of *cytK* expression and regulation.

## 6. Conclusions

In *B. cereus*, a large number of virulence genes are regulated in a cell density dependent manner by activating the expression of the PlcR regulon at the onset of stationary phase [54]. Activation of the *plcR* regulon results in the expression of genes with various functions and the secretion of many proteins that can have a negative effect on host cells, including enterotoxins, zinc metalloproteases, phospholipases, chitinases, collagenases, and two haemolysins: Clo and CytK (Figure 3). This ability to express many different toxins and virulence factors in a coordinated manner could allow the bacteria to increase its pathogenicity [48,55]. Haemolysins, which lyse erythrocytes, leading to the release of nutrients and iron-binding proteins, are among the most widely distributed toxins in pathogenic bacteria. Although it has been clearly established for a variety of other bacterial pathogens that haemolysins belonging to the cholesterol-dependent cytolysin (CDC) family or to the ß-barrel pore-forming toxin family contribute and are sometimes essential for pathogenesis, the exact role of these haemolysins (CLO, HlyII and CytK) in *B cereus* has not been completely elucidated. However, as pore formation is a major threat for cells, the cytotoxic properties of *B. cereus* haemolysins may enable the bacterium to damage epithelial cell layers, such as the intestinal barrier, and gain access to alternative sources of nutrients. In gastrointestinal anthrax, it is hypothesized that ALO may be the primary secreted virulence factor that affects bacterial movement through the epithelial cell barrier for systemic infection after spores are ingested and germinate on or within the epithelium of the gastrointestinal tract [42].

A deletion of plcR results in a strong reduction in virulence against both insect larvae and mice but does not abolish it totally [48], indicating that additional factors not regulated by PlcR contribute to virulence. Other regulators have been shown to also play a role in *B. cereus* virulence. For example, in an insect infection model, the virulence of a ferric iron uptake regulator (*fur*) null strain was found to be significantly attenuated, highlighting the essential role played by Fur in the virulence of this pathogen [81]. HlyII is one of the few known secreted virulence factors of *B. cereus* that does not appear to be regulated by PlcR. Instead, a Fur recognition sequence was found preceding the *hlyII* gene and Fur has been linked to the expression of hemolysin II, along with another gene, *hlyIIR*, subsequently discovered to be *hlylI* regulator [74]. Contrary to CLO and CytK, HlyII is unlikely to be a virulence factor involved in gastrointestinal disease. Instead, it has been shown that HlyII is involved in host macrophages death by apoptosis [63] and, as such, it may help to promote resistance to the host immune system. *In vitro*, the fourth haemolysin, HlyIII, was also shown to be a potent toxin that produces pores within the membrane of susceptible cells [84,86]. However, the *in vivo* toxicity of HlyIII has not yet been established and consequently, its role and contribution in *B. cereus* virulence remains unknown.

An in-depth investigation of the individual and synergistic effect of these toxins in combination with other known virulent determinants is now required. More attention must also be given to the stimuli (such as temperature, PH, oxygen, redox potential, bile salts, and so on) potentially enhancing or reducing toxin activity *in vivo* and to the effects that the human gastro-intestinal tract have on the expression of these secreted virulence factors.
